# Socioeconomic disparities in the mental health of Indigenous children in Western Australia

**DOI:** 10.1186/1471-2458-12-756

**Published:** 2012-09-10

**Authors:** Carrington CJ Shepherd, Jianghong Li, Francis Mitrou, Stephen R Zubrick

**Affiliations:** 1 Centre for Population Health Research, Curtin Health Innovation Research Institute, Curtin University, Perth, Australia; 2 Telethon Institute for Child Health Research, Centre for Child Health Research, University of Western Australia, Perth, Australia

**Keywords:** Socioeconomic, Social disparities, Social gradient, Aboriginal, Mental health, Indigenous, Inequality, Australia

## Abstract

**Background:**

The burden of mental health problems among Aboriginal and Torres Strait Islander children is a major public health problem in Australia. While socioeconomic factors are implicated as important determinants of mental health problems in mainstream populations, their bearing on the mental health of Indigenous Australians remains largely uncharted across all age groups.

**Methods:**

We examined the relationship between the risk of clinically significant emotional or behavioural difficulties (CSEBD) and a range of socioeconomic measures for 3993 Indigenous children aged 4–17 years in Western Australia, using a representative survey conducted in 2000–02. Analysis was conducted using multivariate logistic regression within a multilevel framework.

**Results:**

Almost one quarter (24**%**) of Indigenous children were classified as being at high risk of CSEBD. Our findings generally indicate that higher socioeconomic status is associated with a reduced risk of mental health problems in Indigenous children. Housing quality and tenure and neighbourhood-level disadvantage all have a strong direct effect on child mental health. Further, the circumstances of families with Indigenous children (parenting quality, stress, family composition, overcrowding, household mobility, racism and family functioning) emerged as an important explanatory mechanism underpinning the relationship between child mental health and measures of material wellbeing such as carer employment status and family financial circumstances.

**Conclusions:**

Our results provide incremental evidence of a social gradient in the mental health of Aboriginal and Torres Strait Islander children. Improving the social, economic and psychological conditions of families with Indigenous children has considerable potential to reduce the mental health inequalities within Indigenous populations and, in turn, to close the substantial racial gap in mental health. Interventions that target housing quality, home ownership and neighbourhood-level disadvantage are likely to be particularly beneficial.

## Background

Mental health conditions and disorders are among the leading causes of disability in many countries, and are estimated to account for 13% of the total burden of disease worldwide [1]. The existing epidemiological evidence-base, while limited, confirms that mental health problems are a universal dilemma among children and adolescents, with a global prevalence of about 10–20%, and up to 40% in some low income countries [2].

Mental health disorders have complex aetiologies, with a broad range of factors shown to variably influence them [3] across time and by place and lifecourse stage [4]. Among these factors socioeconomic status (SES) is consistently implicated as an important determinant in both adult [5-9] and child populations [10,11]. Overwhelmingly, quantitative studies show that better SES outcomes are associated with better mental health [12-15]. While this pattern has been observed from early childhood (0–5 years), the association is less consistent among young children, potentially owing to the difficulty in identifying mental illness in children of this age [10].

The theories regarding the mechanisms underpinning the association between SES and mental health are disputed [16,17]. Explanations of SES disparities in mental health tend to support one of two broad hypotheses: that SES factors cause the onset of a mental health condition (social causation), or that poor mental health causes a downward shift in social class or status (health selection). The relative merits of these hypotheses may depend on the outcome of interest [18,19], although both theories support a distal connection between socioeconomic conditions and mental health [20,21].

There are few reliable population-based studies that have specifically aimed to assess the mental health of Indigenous Australians (Aboriginal and Torres Strait Islander peoples; herein referred to as Aboriginal) [22,23]. This partly reflects the difficulties in measuring mental health in culturally distinct populations. The complexities of accurate assessment in these contexts extend to issues of diagnostic validity (e.g. the reliability and validity of mainstream assessment tools, and appropriateness of Western classification systems) [24], misdiagnosis (e.g. as a result of language problems) and under-reporting (e.g. not willing to identify as belonging to a minority group) [25]. These issues are complicated by differences in the definition of mental health concepts and associated terminology between Western and other (including Aboriginal) cultures [23]. The scant quantitative literature, in conjunction with a wider body of qualitative and ethnographic studies, suggests that the mental health outcomes of Aboriginal Australians are particularly poor [25,26], and worse than those of non-Aboriginal Australians [27]. Recent evidence reveals that these disparities are evident in childhood and adolescence [28,29].

The distribution of mental health outcomes across socioeconomic strata within Aboriginal populations of Australia is largely undescribed. A recent review highlighted that the social patterning of physical health in Aboriginal Australia is diverse, and found limited and inconclusive evidence on mental health [30]. While the mental health outcomes of mainstream populations of Australian children typically reflect a social gradient [14,31,32], it is unclear whether this pattern characterises Aboriginal children.

It is plausible that the association between SES and mental health is relatively muted in Aboriginal population groups. It is now well-accepted that the unique post-colonial history of Aboriginal Australia, characterised by widespread dispossession, exclusion, discrimination and marginalisation, has had profoundly negative effects on the wellbeing of Aboriginal peoples. Evidence suggests that these effects include high levels of stress in the lives of a disproportionate number of Aboriginal people in all levels of the social hierarchy [33] and, correspondingly, this may limit the mental health benefits that normally accrue from improved SES. In addition, extended family networks, cultural continuity, and connection to traditional lands may exert a greater influence on Aboriginal health than SES.

Gaining an appreciation of the relationship between SES and the mental health of Aboriginal children is important for a number of reasons. Evidence that details the magnitude and shape of mental health disparities within Aboriginal child populations, and the mechanisms that mediate the impact of SES on mental health, can provide insights into the relative importance of social conditions to child mental health outcomes. This would facilitate a better grasp of the complex underlying mechanisms that lead to poor mental health among Aboriginal children specifically and Aboriginal peoples more generally. It is also likely to broaden the scope of this field of research with the recognition of social factors that may play a critical role in the mental health of Aboriginal children but are not implicated as traditional determinants of mental wellbeing.

Further, there are important policy implications of improving our knowledge in this area. If there are relatively weak socioeconomic gradients in the mental health of Aboriginal child populations then investments aimed at improving socioeconomic conditions (e.g. the employment, income and education of carers) may not translate into the same level of improvement in the mental health of Aboriginal populations as in mainstream populations. Such investments may fail to substantially reduce the disparities in mental health status between Aboriginal and other populations of children. This implies that policy intent, expectations and interventions would need to be modified in order to substantially benefit the mental health of Aboriginal children. Importantly, if interventions can improve the mental health status of Aboriginal children they are likely to have positive consequences for subsequent generations of adults, given that physical and mental wellbeing in childhood builds the foundation for health and development throughout the lifecourse [4,34].

This study aims to examine the nature of the relationship between SES and mental health among Aboriginal children in Western Australia, and the underlying mechanisms, using a rare and large, representative sample that is well-characterised and comprehensively measured. We use a reliable, validated measure of emotional and behavioural difficulties applicable to Aboriginal children and youth in Western Australia [35] to investigate the pattern of associations with conventional and alternative measures of SES at individual, family, household and community levels.

## Methods

Data are from the 2000–2002 Western Australian Aboriginal Child Health Survey (WAACHS), a population representative study of the health, development and education of Aboriginal children aged 0–17 years in the state of Western Australia, and their families and communities. While the data source is now over ten years old, they still provide a reliable assessment of the social, economic and health circumstances of Aboriginal children and families as there have been few significant changes in these circumstances across Australia since the WAACHS data were collected [36]. The survey used an area-based clustered multi-stage sample design. Dwellings in selected census collection districts (CDs) were approached and in-scope families were surveyed, where there was an Aboriginal child aged 0–17 years living in the dwelling. All Aboriginal children aged 0–17 years in in-scope families were selected to participate in the survey. Of all eligible families, 84% consented to participate in the survey and useable information was obtained on 96% of participating children (from interviews with their carers, supplemented with self-reported information from 12–17 year old participants). This netted a final sample of 5289 Aboriginal children living in 1999 responding families, equating to almost 18% of all Aboriginal children living in Western Australia. In addition to data on the health of children, interviews were conducted among primary carers and, where possible, secondary carers of children to gather information on the demographic, social and economic circumstances of families, households and the communities in which they lived. Primary and secondary carers were the people who spent the most time with survey children and knew them best. The primary carer was usually the mother of the child (80%). In the majority of cases, the secondary carer was the father of the child (77%) or another related person (19%). Most primary (83%) and secondary (79%) carers identified themselves as Aboriginal. All aspects of the survey were conducted under the direction of a steering committee of senior Aboriginal people from a cross-section of settings and organisations, to ensure the cultural integrity of survey methods and processes. The full details of the design and conduct of the WAACHS have been described elsewhere [33].

### Measuring mental health

**Figure 1:**
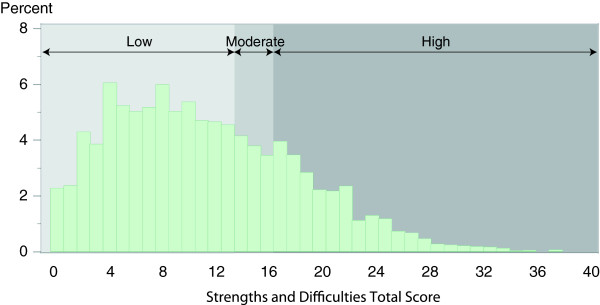
**Distribution of SDQ total scores among aboriginal children aged 4–17 years, Western Australia.** Note: SDQ = Strengths and Difficulties Questionnaire; ‘Low, ‘Moderate’ and ‘High’ indicate risk of clinically significant emotional or behavioural difficulties. Figure from Zubrick et al., 2005, used with permission [29].

Information on mental health outcomes was gathered from primary carers of participating children aged 4–17 years. The Strengths and Difficulties Questionnaire (SDQ) was used to assess risk status for clinically significant emotional or behavioural difficulties (CSEBD) [37,38], and was modified, with permission from the author, to be more suitable for use in Australian Aboriginal populations. Consistent with its design parameters, the SDQ was collected only for participants aged 4–17 years. No reliable indicator of infant and toddler mental health was available to the survey – as such, no mental health data were collected for 0–3 year olds. The 20 questions that examined emotional symptoms, conduct problems, hyperactivity and peer problems were combined to produce a SDQ Total Score (range 0–40). Primary carers’ responses to the SDQ form the basis of the analysis of Aboriginal children’s emotional and behavioural difficulties in this study, with scores of 17–40 indicating that a child was at high risk of CSEBD (Figure 1). The SDQ Total Score demonstrated excellent psychometric properties across a range of geographic areas, from urban to very remote settings (Raykov’s Rho = 0.93) [39]. 

The term ‘mental health’ is used here to describe the WAACHS measure of risk of CSEBD, in preference to ‘social and emotional wellbeing’ (SEWB). Mental health is one aspect of the broader concept of SEWB and its scope does not include the aspects of SEWB that pertain to issues of suicide, self-harm, spiritual wellbeing, and the broader issues that impact on the wellbeing of Aboriginal communities.

### SES measures

SES was measured using seven variables, including characteristics of parents/carers (educational attainment of primary carer and highest occupational class of carers), families/households (family financial strain, housing tenure, housing quality) and neighbourhoods/communities (two composite indexes of socioeconomic disadvantage; one based on the total population and the other on the Aboriginal population only). This array of measures was chosen for four main reasons. First, reliance on a single measure is unlikely to capture how socioeconomic position shapes health disparities in any population. This is particularly true among Aboriginal populations because they are more likely to be distributed at the lower levels of any SES construct [40]. Second, it is necessary to measure different dimensions of SES at multiple levels in order to capture the complex influences of socioeconomic disadvantage on mental health in Aboriginal populations. Third, use of two different area-level SES measures enables us to distinguish compositional from contextual effects on health disparities. Fourth, it is important to test the saliency of conventional versus alternative SES indicators in shaping health disparities, particularly in Aboriginal and other disadvantaged populations [41-43]. We have included conventional indicators of social class (education and occupation) and used a subjective rating of family financial strain as a proxy measure of material wellbeing. Financial strain is used in preference to a conventional measure of household income, for two main reasons: first, income data were not collected from all household members that contributed to its financial base; and second, income does not capture the nature of sharing of economic resources that can occur between members of extended Aboriginal families [44]. Housing characteristics are afforded prominence in these analyses, given that Aboriginal children often experience sub-standard housing that fails to meet the basic requirements for maintaining physical and mental health and social wellbeing [45,46]. Housing tenure and quality can also be considered as proxy indicators of income and wealth [47] and have been included to complement the measure of financial strain (income) in describing the material wellbeing of Aboriginal families and households.

Information about the characteristics of primary carers, families and households was provided by the primary carers of participating children. Secondary carers provided separate responses on their occupational class. Housing quality was measured using a set of indicators based on a nationally agreed framework for the design, construction and maintenance of Indigenous housing [48]. This includes whether the house had facilities for washing people and clothes, removing waste safely, storing and cooking food, and controlling the temperature. Households were classified into one of four categories: having none, one, two, or three or more indicators of poor housing quality.

The Australian Bureau of Statistics’ Socioeconomic Index for Areas (SEIFA) product and Biddle’s Index of Relative Indigenous Socioeconomic Outcomes (IRISEO) were used to measure area-level socioeconomic disadvantage [49,50]. The SEIFA index ranks the relative level of disadvantage of areas using the attributes of all persons (Aboriginal and non-Aboriginal) in each CD, and includes measures of income, educational attainment, employment status and occupational skill. Quintiles were determined based on the distribution of values for all Australian CDs. Biddle’s IRISEO is a rank order variable that measures the socioeconomic outcomes of all 531 Indigenous Areas in Australia in 2001, based on the employment, income, education and housing characteristics of Aboriginal persons only [50]. Quintiles were determined based on the distribution of IRISEO values for all Australian Indigenous Areas.

### Geographic isolation

Geographic isolation is defined using the Level of Relative Isolation (LORI) classification, which is based on the Accessibility/Remoteness Index of Australia (a widely used classification of remoteness in Australia). The five categories of isolation reflect differences in access to services, cultures and health outcomes for Aboriginal children in Western Australia, and range from none (Perth metropolitan area), to low, moderate, high and extreme [51].

### Non-response and imputation

The survey sample was broadly representative of the population of Aboriginal children living in Western Australia, although comparisons with population benchmarks revealed that age, household size and region were significantly associated with non-response. The sample had a lower proportional representation of older children and children living in small households and the south-west region of Western Australia (including the Perth metropolitan area). Post-stratification weighting was employed to adjust for differences in response rates by age, household size and region and produce unbiased estimates. There was only a small amount of non-response to individual questions. While an imputation procedure was employed to assign values to non-responding items, the percentage of imputed values was less than 1% for each variable. Thus, imputation had no effect on the results of this study. Information was unable to be obtained on the characteristics of 15% of secondary carers, and we have treated all variables from these records as missing in the following analysis. More details about non-response characteristics, weighting and imputation are available elsewhere [51].

### Analysis

The analysis in this study was restricted to data from the 3993 children aged 4–17 years for whom the SDQ was collected. Analysis was conducted using logistic regression techniques within a multilevel framework. Models were fitted with the method described by Pfeffermann et al. [52], which takes into account the survey weights and the hierarchical structure of the data, i.e. selection of children within families and communities. A dichotomised total SDQ score was the outcome of interest and modelled separately with each of the following SES variables: carer education, carer occupation, family financial strain, housing tenure, housing quality, SEIFA and IRISEO. Age, sex and LORI are included in the first step (Model 1). Known covariates were entered in blocks at separate steps. The results of successive steps were only reported here if the SES variable achieved marginal statistical significance (p < 0.10). Child physical health factors (whether child had runny ears, whether child had normal vision in both eyes, whether child had difficulty saying certain sounds) were added in the second step (Model 2). Factors related to the physical and mental health of the carer (whether primary carer had a medical condition for 6 months or longer, whether the primary carer had used Mental Health Services) were added in the third step (Model 3). Factors related to the circumstances of the family and household (quality of parenting, life stress events, family composition, overcrowding, number of homes the child had lived in, whether bothered by racism in the neighbourhood/community, and family functioning) were added in the fourth step (Model 4). All models report odds ratios, with the highest status category used as the reference category for ordinal SES variables. Standard errors for survey estimates of total numbers of children were produced using the Ultimate Cluster Variance estimation technique [53]. Standard errors for estimates of odds ratios and proportions were calculated using a modified form of the Jack knife variance estimation technique [54]. Standard chi-square tests adjusted for the complex sample design were used to assess the difference between categorical SES indicators and a dichotomised total SDQ score. SAS version 9.2 was used for all analyses (SAS Institute Inc., Cary, NC, USA, 2000–08).

### Ethical approvals

The WAACHS was conducted under ethical approvals from the (then) Western Australian Aboriginal Health Information and Ethics Committee (WAAHIEC) and the (then) King Edward Memorial and Princess Margaret Hospital Ethics Committee. In addition to the WAAHIEC, this analytic study was approved by Curtin University’s Human Research Ethics Committee and endorsed by the Aboriginal Collaborative Council Advising Research and Evaluation at the Telethon Institute for Child Health Research.

## Results

Almost a quarter (24%) of Aboriginal children was at high risk of clinically significant emotional or behavioural difficulties (CSEBD). Aboriginal children were largely distributed in the more disadvantaged categories of most measures of SES, with few represented in the top category: only 6% of Aboriginal children had a primary carer with a post-secondary education, 5% lived in a family that could ‘save a lot’, and less than 1% lived in areas that fall into the top SEIFA quintile (more advantaged areas). When area-level relative disadvantage based on the characteristics of Aboriginal people only (IRISEO) was analysed, 17% of our study population was in the top two quintiles (Table 1). This signals that, on average, Aboriginal children in Western Australia live in areas with less favourable socioeconomic characteristics than other Aboriginal people across Australia.

**Table 1:** **Mental health, SES and demographic characteristics of Aboriginal children aged 4–17 years in Western Australia**^**a**^

	**Number**	**% (95**% **CI)**
**Mental health status**		
Risk of clinically significant emotional or behavioural difficulties		
Low risk	14800	64.6 (62.2–66.9)
Moderate risk	2610	11.4 (10.3–12.6)
High risk	5490	24.0 (21.9–26.1)
**SES characteristics**		
Education: primary carer		
13 or more years	1370	6.0 (4.6–7.6)
Years 11-12	5080	22.2 (20.0–24.4)
Year 10	9920	43.3 (40.7–46.0)
Year 9 or less^b^	5960	26.0 (23.7–28.4)
Occupation^c^		
Managers and professionals	2910	13.0 (11.2–15.0)
Tradespersons, clerical workers and labourers	8480	38.0 (35.4–40.7)
Not employed	10900	49.0 (46.2–51.8)
Family financial strain		
Can save a lot	1080	4.7 (3.5–6.2)
Can save a bit	5780	25.3 (23.0–27.6)
Some left over but spend it	3040	13.3 (11.5–15.3)
Just enough to get by	10400	45.2 (42.6–47.9)
Spending more than we get	2050	9.0 (7.5–10.6)
Housing tenure		
Owned or being paid off	4800	21.0 (18.6–23.6)
Renting	16600	72.3 (69.6–75.0)
Other	960	4.2 (3.0–5.6)
Number of indicators of poor housing quality		
None	6930	30.3 (27.7–32.9)
One	6180	27.0 (24.7–29.3)
Two	4950	21.6 (19.4–24.0)
Three or more	4840	21.1 (18.9–23.6)
SEIFA^d^ (quintiles)		
Top (more advantaged)	120	0.5 (0.1–1.9)
Third and fourth	3750	16.4 (13.1–20.0)
First and second (less advantaged)	19000	83.1 (79.4–86.5)
IRISEO^e^ (quintiles)		
Top (more advantaged)	260	1.1 (0.4–2.3)
Fourth	3660	16.0 (13.5–18.8)
Third	7310	32.0 (28.9–35.2)
Second	6580	28.8 (25.4–32.4)
First (less advantaged)	5020	22.0 (18.5–25.7)
**Demographics**		
Age (years)		
4-11	13900	60.6 (58.6–62.5)
12-17	9040	39.4 (37.5–41.4)
Sex		
Male	11700	51.2 (49.3–53.1)
Female	11200	48.8 (46.9–50.7)
Level of relative isolation		
None	7830	34.2 (31.6–36.9)
Low	5590	24.4 (21.8–27.1)
Moderate	4680	20.4 (17.1–24.0)
High	2550	11.2 (8.4–14.4)
Extreme	2260	9.8 (7.1–13.0)

^a^ Numbers are weighted estimates of the population of Aboriginal children in each category, and have been rounded. Proportions are based on all Aboriginal children aged 4–17 years (N = 22900). The frequencies of missing responses have not been reported.

^b^ Includes those who had not attended an educational institution.

^c^ Highest occupational class of primary and secondary carers. Occupation categories have been dichotomised based on skill levels defined in the Australian Standard Classification of Occupations, second edition. ‘Managers and professionals’ include occupational skill levels 1 & 2. ‘Tradespersons, clerical workers and labourers’ include occupational skill levels 3–5.

^d^ Customised version of the index of relative socioeconomic disadvantage that forms part of the Australian Bureau of Statistics’ Socioeconomic Index for Areas (SEIFA) product. Quintiles were determined based on the distribution of values for all Australian CDs.

^e^ Biddle’s Index of Relative Indigenous Socioeconomic Outcomes. The index was derived using the characteristics of Aboriginal persons only and quintiles were determined based on the distribution of values for all Australian Indigenous Areas.

Table 2 presents odds ratios from logistic regression analyses, and highlights a generally positive – and significant – association between SES and risk of CSEBD in Aboriginal children, suggesting that those with higher SES have better mental health. The strength and shape of the associations with mental health vary by SES measure, although the most consistent gradients were found for housing quality and tenure. For example, children living in poorer quality housing (three or more indicators of poor quality) were 3.1 times more likely (p < 0.01) to be at high risk of CSEBD than those in the top category (no indicators of poor quality), after adjusting for age, sex and geographic isolation. Children living in rented housing were 1.9 times more likely (p < 0.01) to be at high risk of CSEBD than those in houses that were owned or being paid off by its occupants. The relationship between CSEBD and SEIFA represents a threshold effect, whereby those in the top (most advantaged) SEIFA quintile were at least four times less likely to be at high risk of CSEBD than other children, although only 0.5% of children were in the top quintile (Table 1). While the carer occupation variable was significantly associated with CSEBD, the disparities in odds ratios reflect differences in CSEBD by employment status rather than occupational skill.

**Table 2:** **Relative odds of a mental health problem**^**a**^**, by socioeconomic measure**^**b**^

**Socioeconomic measure**	**Odds ratio: Model 1**^**c**^	**Adjusted odds ratio: Model 2**^**c**^	**Adjusted odds ratio: Model 3**^**c**^	**Adjusted odds ratio: Model 4**^**c**^
Education: primary carer				
13 or more years	1.00			
Years 11–12	1.37	—	—	—
Year 10	1.16			
Year 9 or less^d^	1.81			
Occupation^e^				
Managers/professionals	1.10	1.08	1.07	0.96
Tradespersons, clerical				
workers and labourers	1.00	1.00	1.00	1.00
Not employed	1.94***	1.91***	1.64**	1.17
Family financial strain				
Can save a lot	1.00	1.00	1.00	1.00
Can save a bit	1.75*	1.86**	1.95**	1.56
Some left over but spend it	1.61	1.72*	1.80*	1.25
Just enough to get by	1.79**	1.89**	1.90**	1.23
Spending more than we get	2.70***	2.72***	2.54***	1.34
Housing tenure				
Owned or being paid off	1.00	1.00	1.00	1.00
Renting	1.93***	1.90***	1.83***	1.54***
Other	2.60***	2.55***	2.48***	1.78*
Number of indicators of poor housing quality				
None	1.00	1.00	1.00	1.00
One	1.82**	1.78**	1.52	1.36
Two	2.24***	2.18***	2.02**	1.88**
Three or more	3.13***	2.93***	2.66***	2.80***
SEIFA (quintiles)^f^				
Top (more advantaged)	1.00	1.00	1.00	1.00
Third and fourth	4.81**	4.89**	5.83**	4.43*
First and second (less advantaged)	5.69**	5.91**	6.71**	4.68**
IRISEO (quintiles)^g^				
Top (more advantaged)	1.00			
Fourth	1.82			
Third	1.04	—	—	—
Second	1.58			
First (less advantaged)	0.91			

Notes: *p < 0.1; **p < 0.05; ***p < 0.01; p values are calculated using chi-square tests adjusted for the complex sample design.

^a^ High risk of clinically significant emotional or behavioural difficulties (CSEBD).

^b^ Results are derived from multivariate logistic regression models using a multilevel framework. Results for each SES variable represents a separate model.

^c^ All models include age, sex, Level of Relative Isolation (LORI) and the socioeconomic variable of interest. Model 2 also includes child physical health factors (whether child had runny ears, whether child had normal vision in both eyes, whether child had difficulty saying certain sounds). Model 3 further adds factors related to the physical and mental health of the carer (whether primary carer had a medical condition for 6 months or longer, whether the primary carer had used Mental Health Services). Model 4 further adds factors related to the circumstances of the family and household (quality of parenting, life stress events, family composition, overcrowding, number of homes the child had lived in, whether bothered by racism in the neighbourhood/community, and family functioning). Successive steps were conducted if the socioeconomic variable achieved marginal statistical significance (p < 0.1).

^d^ Includes those who had not attended an educational institution.

^e^ Highest occupational class of primary and secondary carers. Occupation categories have been dichotomised based on skill levels defined in the Australian Standard Classification of Occupations, second edition. ‘Managers and professionals’ include occupational skill levels 1 & 2. ‘Tradespersons, clerical workers and labourers’ include occupational skill levels 3–5.

^f^ Customised version of the index of relative socioeconomic disadvantage that forms part of the Australian Bureau of Statistics’ Socioeconomic Index for Areas (SEIFA) product. Percentiles were determined based on the distribution of values for all Australian CDs.

^g^ Biddle’s Index of Relative Indigenous Socioeconomic Outcomes. The index was derived using the characteristics of Aboriginal persons only and quintiles were determined based on the distribution of values for all Australian Indigenous Areas.

There was a positive, but not continuous, gradient between the primary carer’s educational level and the child’s mental health, although the effects were not statistically significant. There was no clear pattern in CSEBD outcomes when using IRISEO as the SES indicator.

**Figure 2:**
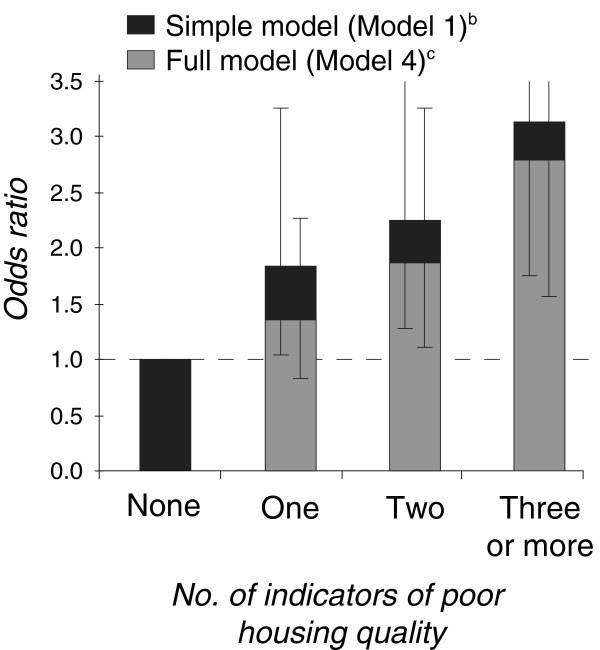
**Relative odds of a mental health problem**^**a**^**, by number of indicators of poor housing quality.**^a^ High risk of clinically significant emotional or behavioural difficulties. ^b^ Simple model (Model 1) adjusts for age, sex and geographic isolation. ^c^ Full model (Model 4) also adjusts for a range of factors related to the physical health of the child, the physical and mental health of the carer, and the circumstances of the family and household.

The relationships between SES and CSEBD are partly attenuated by other known covariates – especially by factors that describe the circumstances of Aboriginal families and households, such as parenting quality, life stress events, family composition, overcrowding, household mobility, perceptions of racism in the neighbourhood, and family functioning. This is most evident for occupation and family financial strain, where adjusted effect sizes are reduced to close to null (Table 2). In contrast, the inclusion of covariates describing aspects of the physical health of the child had little impact on the strength of the social gradients in mental health, whereas the physical and mental health of the carer had a modest influence on the relationships between mental health and occupation, family financial strain and housing quality (Table 2). Housing quality, housing tenure and SEIFA continue to be strongly associated with Aboriginal child mental health after adjusting for the full range of relevant covariates available from the dataset, although there is some attenuation of the odds ratios in the case of the latter two variables (Table 2 and Figure 2).

Additional file 1, Additional file 2 and Additional file 3 provide separate odds ratios for all variables (SES and other known covariates) in logistic regression models where carer occupation, family financial strain and housing tenure are the primary independent variable of interest, respectively. They highlight independent significant associations between CSEBD and all of the included covariates. The results affirm that children have an elevated odds of CSEBD if they had experienced runny ears, vision problems or difficulty saying certain sounds, had a primary carer that had used Mental Health Services or had a chronic medical problem, lived in a sole parent family or without a biological parent, experienced poor parenting quality, poor family functioning, significant life stress or racism, or had moved homes a lot. In contrast, being female, an older child, or living in overcrowded conditions or in the most isolated areas, appeared to be protective of mental health. Among these variables, the strongest associations with CSEBD were found with quality of parenting, life stress events, geographic isolation and whether the child had difficulty saying certain sounds – with odds ratios typically exceeding 3.

## Discussion

The pervasive inequalities in health between Aboriginal and non-Aboriginal people in Australia has demanded a better understanding of the aetiology of poor health outcomes in Aboriginal populations – including mental health. While the current scientific literature implicates social factors and processes in the complex pathways to mental health problems, there has been little scrutiny of the saliency of these factors in Aboriginal population groups.

Our findings generally indicate that higher SES is associated with a reduced risk of clinically significant emotional or behavioural difficulties (mental health problems) in Aboriginal children. Housing and neighbourhood SES characteristics feature prominently in this study, with housing tenure, housing quality and neighbourhood-level disadvantage all having a strong direct effect on mental health. These results are consistent with the extant literature that acknowledges the multiple benefits of housing and neighbourhoods to mental wellbeing [46,55-58]. Previous research has shown that housing has indirect effects on mental health via material and psychosocial pathways. For example, inadequate housing can lead to social disruption and stress and can limit access to services, while home ownership generally provides greater control over the living environment and choice of neighbourhood [47]. The relatively high prevalence of inadequate housing among Aboriginal peoples, the unique geographic dispersion of Aboriginal populations, and the added difficulties in providing and maintaining quality housing in remote communities, may add to the significance of housing as a critical determinant of the mental health of Aboriginal children.

The circumstances of Aboriginal families and households emerged as an important explanatory mechanism, particularly in the relationship between child mental health and both carer employment status and family financial circumstances. This suggests that factors such as parenting quality, stress, family composition, overcrowding, residential mobility, racism and family functioning have a substantial mediating role in the pathway from material wellbeing to poor mental health. Stress is of particular importance here as it has been shown to be a feature of the lives of many Aboriginal families [57,59,60], and to have deleterious effects on the developing brain, including emotional functioning [61]. Racism and overcrowded living conditions are two of the key sources of stress faced by Aboriginal people and families and have been shown to exacerbate mental health problems [56,62]. Overcrowding has been cited as a common problem in households with Aboriginal people [63] – particularly in remote communities [64]– and can magnify stress in a number of ways. More household residents can lead to less privacy, increased noise, lack of sleep, and a general loss of control. It can also increase contact between residents, which has been shown to promote the spread of infection and disease [57,65,66] and, accordingly, increase the strain and anxiety in a person’s life. Racism occurs at both interpersonal and systemic levels in Australian society and it impacts a disturbingly high proportion of Aboriginal people [67]. While the effects of racism on Aboriginal wellbeing is an emerging area of research in Australia, the international literature suggests that discrimination and racism may be a direct cause of psychological distress and/or have an indirect effect on wellbeing via pathways involving smoking and alcohol and substance misuse [68].

While stress is consistently implicated as a primary link between SES and mental health [7,8,10,11], most of the hypothesised pathways have not been fully or adequately investigated in child populations [10]. It is also plausible that stress, racism and overcrowding (and the other potential mediators discussed above) lead to lower SES which, in turn, has a detrimental impact on mental wellbeing. For example, interpersonal and systemic racism can limit the labour market opportunities of parents, leading to a range of stresses that stem from financial insecurity.

The lack of clear evidence of a relationship between primary carer education and child mental health is notable, considering the substantial body of literature that highlights the positive impact of parental education – particularly that of the mother – on child development and wellbeing [4,69,70]. This finding however is consistent with results on aspects of the physical health (scabies, respiratory and ear infections, and diarrhoea and vomiting) of Aboriginal children in remote settings in the Northern Territory of Australia,[57] and may reflect Aboriginal peoples’ often adverse interactions with mainstream Australia since colonisation and the associated legacies. For instance, Western education systems have been heavily implicated in the policies and practices of forced separation from family and kinship networks that were a widespread phenomenon in Australia until the 1970s [71]. The removal of children into missions and other institutions may have provided more formal education for some but had profound detrimental effects on the psychosocial functioning of these “stolen generation” children and their onward ability to adequately undertake the tasks of parenthood [72,73]. Discrimination and racism is a common thread to past practices of dispossession and removal and the persistent marginalisation of Aboriginal peoples’ in present day Australian society. Racism has been shown to limit the ability of parents to promote optimal child development, by increasing psychological distress and disrupting community cohesion and the supports for raising children [74]. These stresses are likely to impair the ability of all parents to cope and could plausibly overwhelm the protective effects of parental education on child mental wellbeing.

### Strengths and limitations

The main strengths of this study are that it: (1) draws upon a large and representative dataset that was collected using robust and culturally appropriate methods and processes; (2) utilises a validated and reliable tool for assessing mental health problems; (3) employs rigorous analytical methods; and (4) uses a wide range of SES indicators that measure different aspects of socioeconomic disadvantage in the Western Australian Aboriginal population.

The main limitation is our reliance on cross-sectional data which limits our ability to assess the causal relationships between SES and mental health. Further, a range of generic and context-specific difficulties in measuring SES may have influenced our results. First, SES may have been incorrectly reported by some survey participants. Some participants may have considered expenditure on wealth creation initiatives (e.g., home loan repayments) as a family financial strain. If this interpretation was consistently applied by participants then financial strain will be overstated and potentially lessen the strength of mental health disparities for this SES measure. Second, there are difficulties in creating robust and meaningful SES measures in Aboriginal contexts. For instance, standard indicators of educational attainment typically ignore knowledge that is valued in Indigenous society (that may have an impact on wellbeing) but acquired outside of Western education systems. Third, our measure of education attainment does not capture the quality of the educational experiences of carers. The relatively poor performance of Aboriginal people in education is well-documented [36,75], and suggests that, at every level of education, Aboriginal people may acquire less health-benefitting knowledge and skills than non-Aboriginal people. If this is applicable to our study sample then we are likely to have understated the strength of the association between carer education and mental health. Fourth, our IRISEO measure is constructed using relatively broad geographic areas where the Aboriginal population often constitute a small minority; consequently, the index may mask the SES characteristics of the total population of an area, and variations in SES within areas. In addition, IRISEO does not capture all community-level SES variables or the full spectrum of factors that have been identified by Aboriginal Australians as important to community wellbeing, such as the resources gained from traditional subsistence activities, access to traditional lands and cultural maintenance [50]. Accordingly, the lack of a clear association between child mental health and the area-level SES characteristics of the Aboriginal population may be an artefact of the composition of the IRISEO measure.

## Conclusions

Our findings are consistent with the prevailing pattern in the mainstream literature – in Australia and elsewhere – where higher parental and household SES is generally associated with better child mental health outcomes [31,32,70,76-80]. This study, in conjunction with a small set of studies of Aboriginal child, youth and adult populations in Australia [27,81-83], provides incremental evidence of a social gradient in the mental health of Aboriginal populations. This has important policy implications, particularly in light of the considerably higher prevalence of mental health problems among Aboriginal children than non-Aboriginal children in Western Australia [29]. The larger burden of mental health among Aboriginal children represents a major public health problem affecting Australian society as a whole. Our findings suggest that improving the social, economic and psychological conditions of Aboriginal families has considerable potential to reduce the mental health inequalities within Aboriginal populations and, in turn, to close the substantial racial gap in mental health. Interventions that target housing quality, home ownership and neighbourhood-level disadvantage are likely to be particularly beneficial. Part of the goal should be to reduce the number of life stresses faced by Aboriginal families, which is likely to have significant payoffs for Aboriginal child wellbeing and development.

## Competing interests

The authors declare that they have no competing interests.

## Authors’ contributions

CCJS was the primary author of the manuscript, completed the analysis of data and interpretation of results. JL conceived of the study and assisted with writing the manuscript. FM assisted with data analysis and edited the manuscript. SRZ conceived of the study and edited the manuscript. All authors read and approved the final manuscript.

## Pre-publication history

The pre-publication history for this paper can be accessed here:

http://www.biomedcentral.com/1471-2458/12/756/prepub

## Supplementary Material

Additional file 1 **Relative odds of a mental health problem, by carer occupation and factors related to the child’s physical health, the physical and mental health of the carer, and the circumstances of the family and household.** Odds ratios from logistic regression analyses are provided for the primary explanatory variable (carer occupation) and separately for a range of known covariates with mental health. Click here for file

Additional file 2 **Relative odds of a mental health problem, by family financial strain and factors related to the child’s physical health, the physical and mental health of the carer, and the circumstances of the family and household.** Odds ratios from logistic regression analyses are provided for the primary explanatory variable (family financial strain) and separately for a range of known covariates with mental health. Click here for file

Additional file 3 **Relative odds of a mental health problem, by housing tenure and factors related to the child’s physical health, the physical and mental health of the carer, and the circumstances of the family and household.** Odds ratios from logistic regression analyses are provided for the primary explanatory variable (housing tenure) and separately for a range of known covariates with mental health. Click here for file

## References

[B1] World Health Organization Mental health and development: targeting people with mental health conditions as a vulnerable group 2010 Geneva: World Health Organization

[B2] Kieling C Baker-Henningham H Belfer M Conti G Ertem I Omigbodun O Rohde LA Srinath S Ulkuer N Rahman A Global mental health 2 child and adolescent mental health worldwide: evidence for action Lancet 2011 378 9801 1515 1525 10.1016/S0140-6736(11)60827-1 22008427

[B3] Susser ES Psychiatric epidemiology: searching for the causes of mental disorders 2006 Oxford University Press, New York

[B4] Keating DP Hertzman C Developmental health and the wealth of nations: Social, Biological, and Educational Dynamics 1999 New York: Guilford Press

[B5] Dohrenwend BP Socioeconomic status (SES) and psychiatric disorders Soc Psychiatry Psychiatr Epidemiol 1990 25 1 41 47 2406949 10.1007/BF00789069

[B6] Anderson NB Armstead CA Toward understanding the association of socioeconomic status and health: a new challenge for the biopsychosocial approach Psychosom Med 1995 57 3 213 225 7652122 10.1097/00006842-199505000-00003

[B7] Matthews KA Gallo LC Taylor SE Are psychosocial factors mediators of socioeconomic status and health connections? Ann N Y Acad Sci 2010 1186 1 146 173 10.1111/j.1749-6632.2009.05332.x 20201872

[B8] Kessler RC Cleary PD Social class and psychological distress Am Sociol Rev 1980 45 3 463 478 10.2307/2095178 7406359

[B9] Adda J Chandola T Marmot M Socio-economic status and health: causality and pathways J conometrics 2003 112 1 57 63

[B10] Bradley RH Corwyn RF Socioeconomic status and child development Annu Rev Psychol 2002 53 371 10.1146/annurev.psych.53.100901.135233 11752490

[B11] Currie J Healthy, wealthy, and wise: socioeconomic status, poor health in childhood, and human capital development J Econ Lit 2009 47 1 87 122 10.1257/jel.47.1.87

[B12] Muntaner C Eaton WW Miech R O'Campo P Socioeconomic position and major mental disorders Epidemiol Rev 2004 26 1 53 62 10.1093/epirev/mxh001 15234947

[B13] Lorant V Deliege D Eaton W Robert A Philippot P Ansseau M Socioeconomic inequalities in depression: A meta-analysis Am J Epidemiol 2003 157 2 98 112 10.1093/aje/kwf182 12522017

[B14] Glover J Hetzel D Tennant S The socioeconomic gradient and chronic illness and associated risk factors in Australia Aust New Zealand Health Policy 2004 1 1 8 10.1186/1743-8462-1-8 15679942 PMC546403

[B15] Abas M Vanderpyl J Robinson E Crampton P More deprived areas need greater resources for mental health Aust N Z J Psychiatry 2003 37 437 444 10.1046/j.1440-1614.2003.01206.x 12873328

[B16] Adler NE Snibbe AC The role of psychosocial processes in explaining the gradient between socioeconomic status and health Curr Dir Psychol 2003 12 4 119 123 10.1111/1467-8721.01245

[B17] Lund C Breen A Flisher AJ Kakuma R Corrigall J Joska JA Swartz L Patel V Poverty and common mental disorders in low and middle income countries: A systematic review Soc Sci Med 2010 71 3 517 528 10.1016/j.socscimed.2010.04.027 20621748 PMC4991761

[B18] West P Rethinking the health selection explanation for health inequalities Soc Sci Med 1991 32 4 373 384 10.1016/0277-9536(91)90338-D 2024152

[B19] Dohrenwend B Levav I Shrout P Schwartz S Naveh G Link B Skodol A Stueve A Socioeconomic status and psychiatric disorders: the causation-selection issue Science 1992 255 5047 946 952 10.1126/science.1546291 1546291

[B20] Link BG Phelan J Social conditions as fundamental causes of disease J Health Soc Behav 1995 35 80 94 7560851

[B21] Page AN Swannell S Martin G Hollingworth S Hickie IB Hall WD Sociodemographic correlates of antidepressant utilisation in Australia Med J Aust 2009 190 9 479 483 19413517 10.5694/j.1326-5377.2009.tb02522.x

[B22] Zubrick SR D'Antoine H The WAACHS Team Fitzgerald HE, Puura K, Tomlinson M, Paul C The mental health of Australian aboriginal children and adolescents: current status and future prospects International Perspectives on Children and Mental Health: Volume 2 Prevention and Treatment 2011 ABC-CLIO, Santa Barbara

[B23] Garvey D A review of the social and emotional wellbeing of indigenous Australian peoples - considerations, challenges and opportunities Aust Indigenous HealthBulletin 2008 8 3 1 29

[B24] Cohen A The mental health of indigenous peoples: an international overview 1999 Nations for Mental Health, Department of Mental Health, World Health Organization, Geneva

[B25] Anderson I Baum F Bentley M Beyond Bandaids: Exploring the Underlying Social Determinants of Aboriginal Health. Papers from the Social Determinants of Aboriginal Health Workshop, Adelaide, July 2004 2007 Cooperative Research Centre for Aboriginal Health, Darwin

[B26] Hunter E Disadvantage and discontent: a review of issues relevant to the mental health of rural and remote indigenous Australians Aust J Rural Health 2007 15 2 88 93 10.1111/j.1440-1584.2007.00869.x 17441816

[B27] Australian Health Ministers' Advisory Council Aboriginal and Torres Strait Islander Health Performance Framework Report 2011 Australian Health Ministers' Advisory Council, Canberra

[B28] Australian Institute of Health and Welfare A picture of Australia’s children 2009 2009 Australian Institute of Health and Welfare, Canberra

[B29] Zubrick SR Silburn SR Lawrence DM Mitrou FG Dalby RB Blair EM Griffin J Milroy H De Maio JA Cox A Li J The Western Australian Aboriginal Child Health Survey: The Social and Emotional Wellbeing of Aboriginal Children and Young People 2005 Telethon Institute for Child Health Research, Perth

[B30] Shepherd CCJ Li J Zubrick SR Social gradients in the health of indigenous australians Am J Public Health 2012 102 1 107 117 10.2105/AJPH.2011.300354 22095336 PMC3490556

[B31] Davis E Sawyer MG Lo SK Priest N Wake M Socioeconomic risk factors for mental health problems in 4-5-year-old children: Australian population study Acad Pediatr 2010 10 1 41 47 10.1016/j.acap.2009.08.007 20129480

[B32] Sawyer MG Arney FM Baghurst PA Clark JJ Graetz BW Kosky RJ Nurcombe B Patton GC Prior MR Raphael B Rey J Whaites LC Zubrick SR Mental health of young people in Australia 2000 Mental Health and Special Programs Branch, Commonwealth Department of Health and Aged Care, Canberra

[B33] Silburn SR Zubrick SR De Maio JA Shepherd C Griffin JA Mitrou FG Dalby RB Hayward C Pearson G The Western Australian Aboriginal Child Health Survey: Strengthening the Capacity of Aboriginal Children, Families and Communities 2006 Telethon Institute for Child Health Research, Perth

[B34] McCain M Mustard JF Early years study: Reversing the real brain drain 1999 Ontario Children’s Secretariat, Toronto, ON

[B35] De Maio JA Zubrick SR Silburn SR Lawrence DM Mitrou FG Dalby RB Blair EM Griffin J Milroy H Cox A The Western Australian Aboriginal Child Health Survey: measuring the social and emotional wellbeing of Aboriginal children and the intergenerational effects of forced separation 2005 Curtin University of Technology and Telethon Institute for Child Health Research, Perth

[B36] Steering Committee for the Review of Government Service Provision (SCRGSP) Overcoming Indigenous disadvantage: Key indicators 2011 2011 Productivity Commission, Canberra

[B37] Goodman R The extended version of the strengths and difficulties questionnaire as a guide to child psychiatric caseness and consequent burden J Ch Psychol Psychiat 1999 40 5 791 799 10.1111/1469-7610.00494 10433412

[B38] Goodman R Ford T Simmons H Gatward R Meltzer H Using the strengths and difficulties questionnaire (SDQ) to screen for child psychiatric disorders in a community sample Br J Psychiatry 2000 177 6 534 539 10.1192/bjp.177.6.534 11102329

[B39] Zubrick S Lawrence D De Maio J Biddle N Testing the reliability of a measure of Aboriginal children's mental health: An analysis based on the Western Australian Aboriginal Child Health Survey 2006 Telethon Institute for Child Health Research & Australian Bureau of Statistics, Perth

[B40] Shepherd CCJ Li J Zubrick SR Socioeconomic disparities in physical health among aboriginal and Torres Strait islander children in Western Australia Ethn Health 2012 1 23 22292856 10.1080/13557858.2012.654768

[B41] Shavers VL Measurement of socioeconomic status in health disparities research J Natl Med Assoc 2007 99 9 1013 1023 17913111 PMC2575866

[B42] Taylor J Indigenous peoples and indicators of well-being: Australian perspectives on United Nations global frameworks Soc Indic Res 2008 87 1 111 126 10.1007/s11205-007-9161-z

[B43] Altman JC The economic status of Indigenous Australians. CAEPR Discussion Paper No. 193 2000 The Centre for Aboriginal Economic Policy Research, The Australian National University, Canberra

[B44] Hunter B Kennedy S Smith D Household composition, equivalence scales and the reliability of income distributions: some evidence for indigenous and other Australians Econ Rec 2003 79 244 70 83 10.1111/1475-4932.00079

[B45] Bailie RS Runcie MJ Household infrastructure in Aboriginal communities and the implications for health improvement Med J Aust 2001 175 7 363 366 11700813 10.5694/j.1326-5377.2001.tb143619.x

[B46] Bailie R Carson B, Dunbar T, Chenhall RD, Bailie R Housing Social Determinants of Indigenous Health 2007 Allen & Unwin, Sydney 203 230

[B47] Shaw M Housing and public health Annu Rev Public Health 2004 25 397 418 10.1146/annurev.publhealth.25.101802.123036 15015927

[B48] Commonwealth State and Territory Housing Ministers’ Working Group on Indigenous Housing National framework for the design, construction and maintenance of Indigenous housing — Incorporating the National Indigenous Housing Guide 1999 Commonwealth Department of Family and Community Services, Canberra

[B49] Australian Bureau of Statistics Information Paper: 1996 Census of Population and Housing. Socioeconomic Index for Areas 1998 Australian Bureau of Statistics, Canberra

[B50] Biddle N Ranking Regions: Revisiting an Index of Relative Indigenous Socioeconomic Outcomes 2009 Centre for Aboriginal Economic Policy Research, The Australian National University, Canberra

[B51] Zubrick SR Lawrence DM Silburn SR Blair E Milroy H Wilkes T Eades S D’Antoine H Read A Ishiguchi P Doyle S Western Australian Aboriginal child health survey: The Health of Aboriginal Children and Young People 2004 Telethon Institute for Child Health Research, Perth

[B52] Pfeffermann D Skinner CJ Holmes DJ Goldstein H Rasbash J Weighting for unequal selection probabilities in multilevel models J R Stat Soc Series B Stat Methodol 1998 60 1 23 40 10.1111/1467-9868.00106

[B53] Wolter KM Introduction to Variance Estimation 1985 Springer Verlag, New York

[B54] Jones HL Jackknife estimation of functions of stratum means Biometrika 1974 61 2 343 348

[B55] Diez Roux AV Mair C Neighborhoods and health Ann N Y Acad Sci 2010 1186 1 125 145 10.1111/j.1749-6632.2009.05333.x 20201871

[B56] Bailie RS Wayte KJ Housing and health in indigenous communities: key issues for housing and health improvement in remote aboriginal and Torres Strait islander communities Aust J Rural Health 2006 14 5 178 183 10.1111/j.1440-1584.2006.00804.x 17032292

[B57] Bailie R Stevens M McDonald E Brewster D Guthridge S Exploring cross-sectional associations between common childhood illness, housing and social conditions in remote Australian aboriginal communities BMC Public Health 2010 10 1 147 10.1186/1471-2458-10-147 20302661 PMC2848201

[B58] Priest N Paradies Y Stevens M Bailie R Exploring relationships between racism, housing and child illness in remote indigenous communities J Epidemiol Community Health 2012 66 5 440 447 10.1136/jech.2010.117366 21118951

[B59] Swan P Raphael B Ways Forward: National Aboriginal and Torres Strait Islander Mental Health Policy National Consultancy Report 2004 Australian Government Publishing Service, Canberra

[B60] Kowal E Gunthorpe W Bailie R Measuring emotional and social wellbeing in aboriginal and Torres Strait islander populations: an analysis of a negative life events scale Int J Equity Health 2007 6 1 1861 10.1186/1475-9276-6-18 PMC2203968 18001479

[B61] McEwen BS Early life influences on life-long patterns of behavior and health Ment Retard Dev Disabil Res Rev 2003 9 3 149 154 10.1002/mrdd.10074 12953293

[B62] Priest NC Paradies YC Gunthorpe W Cairney SJ Sayers SM Racism as a determinant of social and emotional wellbeing for aboriginal Australian youth Med J Aust 2011 194 10 546 550 21644910 10.5694/j.1326-5377.2011.tb03099.x

[B63] Australian Bureau of Statistics, Australian Institute of Health and Welfare Housing circumstances: overcrowding. In: The Health and Welfare of Australia's Aboriginal and Torres Strait Islander Peoples 2010 Australian Bureau of Statistics and Australian Institute of Health and Welfare, Canberra

[B64] Bailie RS McDonald EL Stevens M Guthridge S Brewster DR Evaluation of an Australian indigenous housing programme: community level impact on crowding, infrastructure function and hygiene J Epidemiol Community Health 2011 65 5 432 437 10.1136/jech.2009.091637 20466712 PMC3071088

[B65] Bailie RS Stevens M McDonald EL The impact of housing improvement and socio-environmental factors on common childhood illnesses: a cohort study in indigenous Australian communities J Epidemiol Community Health 2012 66 9 821 831 10.1136/jech.2011.134874 21693472 PMC3412050

[B66] McDonald E Bailie R Grace J Brewster D An ecological approach to health promotion in remote Australian aboriginal communities Health Promot Int 2010 25 1 42 53 10.1093/heapro/daq004 20167824

[B67] Paradies Y Harris R Anderson I The Impact of Racism on Indigenous Health in Australia and Aotearoa: Towards a Research Agenda 2008 Cooperative Research Centre for Aboriginal Health, Darwin

[B68] Paradies Y A systematic review of empirical research on self-reported racism and health Int J Epidemiol 2006 35 4 888 901 10.1093/ije/dyl056 16585055

[B69] Cochrane SH Leslie J Ohara DJ Parental education and child health - intracountry evidence Health Policy Educ 1982 2 3–4 213 250 10298649 10.1016/0165-2281(82)90011-x

[B70] Carneiro P Meghir C Parey M Maternal Education, Home Environments and the Development of Children and Adolescents 2007 University College London and Institute for Fiscal Studies, London

[B71] Boughton B What is the connection between Aboriginal education and Aboriginal health? Occasional paper no.2 2000 2001 Cooperative Research Centre for Aboriginal and Tropical Health, Casuarina

[B72] Beresford Q Partington G Reform and resistance in Aboriginal education: The Australian experience 2003 University of Western Australia Press, Crawley

[B73] Human Rights and Equal Opportunity Commission Bringing Them Home: National Inquiry into the Separation of Aboriginal and Torres Strait Islander Children from Their Families 1997 Human Rights and Equal Opportunity Commission, Canberra

[B74] Sanders-Phillips K Racial discrimination: a continuum of violence exposure for children of color Clin Child Fam Psychol Rev 2009 12 2 174 195 10.1007/s10567-009-0053-4 19466544

[B75] Zubrick SR Silburn SR De Maio JA Shepherd C Griffin JA Dalby RB Mitrou FG Lawrence DM Hayward C Pearson G Milroy H Milroy J Cox A The Western Australian Aboriginal Child Health Survey: Improving the Educational Experiences of Aboriginal Children and Young People 2006 Telethon Institute for Child Health Research, Perth

[B76] McLeod JD Shanahan MJ Poverty, parenting, and childrens mental-health Am Sociol Rev 1993 58 3 351 366 10.2307/2095905

[B77] Berger LM Paxson C Waldfogel J Income and child development Chil Youth Ser R 2009 31 9 978 989 10.1016/j.childyouth.2009.04.013 PMC2847736 20368763

[B78] Larson K Halfon N Family income gradients in the health and health care access of us children Matern Child Health J 2010 14 3 332 342 10.1007/s10995-009-0477-y 19499315 PMC2862175

[B79] Spencer N Social, economic, and political determinants of child health Pediatrics 2003 112 3 704 706 12949325

[B80] Xue Y Leventhal T Brooks-Gunn J Earls FJ Neighborhood residence and mental health problems of 5- to 11-year-olds Arch Gen Psychiatry 2005 62 5 554 563 10.1001/archpsyc.62.5.554 15867109

[B81] Priest N Paradies Y Stewart P Luke J Racism and health among urban aboriginal young people BMC Public Health 2011 11 1 568 10.1186/1471-2458-11-568 21756369 PMC3146875

[B82] Larson A Gillies M Howard PJ Coffin J It's enough to make you sick: the impact of racism on the health of Aboriginal Australians Aust N Z J Public Health 2007 31 4 322 329 10.1111/j.1753-6405.2007.00079.x 17725009

[B83] Ziersch A Gallaher G Baum F Bentley M Racism, social resources and mental health for aboriginal people living in Adelaide Aust N Z J Public Health 2011 35 3 231 237 10.1111/j.1753-6405.2011.00681.x 21627723

