# Supplementary material for: Socioeconomic disparities in the mental health of Indigenous children in Western Australia
Source: BMC Public Health. 2012 Sep 10;12:756. doi: 10.1186/1471-2458-12-756 (PMC3508977; doi:10.1186/1471-2458-12-756)
Supplement: Additional file 3 — Relative odds of a mental health problem, by housing tenure and factors related to the child’s physical health, the physical and mental health of the carer, and the circumstances of the family and household. Odds ratios from logistic regression analyses are provided for the primary explanatory variable (housing tenure) and separately for a range of known covariates with mental health. [file 1471-2458-12-756-S3.doc]

## Additional file 3 - Relative odds of a mental health problema, by housing tenure and factors related to the child’s physical health, the physical and mental health of the carer, and the circumstances of the family and householdb

| **Socioeconomic and other measures** | **Odds ratio: Model 1^c^** | **Adjusted odds ratio: Model 2^c^** | **Adjusted odds ratio: Model 3^c^** | **Adjusted odds ratio: Model 4^c^** |
| --- | --- | --- | --- | --- |
| Housing tenure  Owned or being paid off  Renting  Other | 1.00  1.93***  2.60*** | 1.00  1.90***  2.55*** | 1.00  1.83***  2.48*** | 1.00  1.54***  1.78* |
| Age  4  5  6  7  8  9  10  11  12  13  14  15  16  17 | 1.23  1.00  1.37  1.22  1.24  1.24  1.09  0.80  1.26  1.26  1.10  0.58**  0.75  0.63 | 1.30  1.00  1.53**  1.50*  1.44*  1.45*  1.32  0.96  1.55**  1.53*  1.41  0.74  0.96  0.82 | 1.28  1.00  1.49**  1.46*  1.39  1.43*  1.30  0.93  1.49*  1.49*  1.29  0.69  0.87  0.74 | 1.31  1.00  1.42  1.45  1.34  1.23  1.28  0.86  1.31  1.45  1.19  0.59*  0.79  0.59* |
| Sex  Males  Females | 1.00  0.65*** | 1.00  0.67*** | 1.00  0.66*** | 1.00  0.64*** |
| Level of relative isolation  None (Perth metropolitan area)  Low  Moderate  High  Extreme | 1.00  0.80  0.91  0.70  0.29*** | 1.00  0.79*  0.94  0.71  0.29*** | 1.00  0.77*  0.91  0.74  0.29*** | 1.00  0.81  0.86  0.90  0.34*** |
| Whether child had runny ears^d^  No  Yes |  | 1.00  1.57*** | 1.00  1.55*** | 1.00  1.41*** |
| Whether child had normal vision in both eyes  No  Yes |  | 1.66***  1.00 | 1.60***  1.00 | 1.50**  1.00 |
| Whether child had difficulty saying certain sounds  No  Yes |  | 0.42***  1.00 | 0.43***  1.00 | 0.45***  1.00 |
| Whether the primary carer had used Mental Health Services^e^  No  Yes  Don’t know |  |  | 1.00  1.54***  1.12 | 1.00  1.32**  1.15 |
| Whether primary carer had a medical condition for 6 months or longer  No  Yes |  |  | 1.00  1.61*** | 1.00  1.63*** |
| Quality of parenting^f^  Very good  Good  Fair  Poor |  |  |  | 0.44***  0.52***  0.65***  1.00 |
| Family composition  Two parent family  Sole parent  Two parent step/blended  Other (e.g. Aunts/uncles) |  |  |  | 1.00  1.53***  1.06  1.92*** |
| Overcrowding^g^  Household occupancy level – Low  Household occupancy level – High |  |  |  | 1.00  0.63*** |
| Number of homes the child had lived in  1-4 homes  5 or more homes |  |  |  | 0.80**  1.00 |
| Family functioning^h^  Poor  Fair  Good  Very good |  |  |  | 1.38*  1.00  1.14  0.83 |
| Life stress events^i^  0–2  3–4  5–6  7–14 |  |  |  | 0.72*  1.00  1.04  2.04*** |
| Whether bothered by racism in the neighbourhood/community  No  Yes |  |  |  | 1.00  1.40*** |

Notes: *p < 0.1; **p < 0.05; ***p < 0.01; p values are calculated using chi-square tests adjusted for the complex sample design.

^a^ High risk of clinically significant emotional or behavioural difficulties (CSEBD).

^b^ Results are derived from multivariate logistic regression models using a multilevel framework.

^c^ All models include age, sex, Level of Relative Isolation (LORI) and housing tenure. Model 2 also includes child physical health factors (whether child had runny ears, whether child had normal vision in both eyes, whether child had difficulty saying certain sounds). Model 3 further adds factors related to the physical and mental health of the carer (whether primary carer had a medical condition for 6 months or longer, whether the primary carer had used Mental Health Services). Model 4 further adds factors related to the circumstances of the family and household (quality of parenting, life stress events, family composition, overcrowding, number of homes the child had lived in, whether bothered by racism in the neighbourhood/community, and family functioning). Successive steps were conducted if the socioeconomic variable achieved marginal statistical significance (p < 0.1).

^d^ A discharge from the ear as a result of an eardrum rupture (usually from otitis media, or infection of the middle ear).

^e^ Contact with Mental Health Services in Western Australia.

^f^ An index of quality of parenting derived from responses to three items: how often carers praise their children, how often they hit or smack their children and how often they laugh together with their children. These items were rated by carers on a five-point frequency scale from ‘Never’ through to ‘Almost always’. An overall score was produced by summing these three items. Scores were ranked and categorising into quartiles, and labelled ‘poor’, ‘fair’, ‘good’ and ‘very good’.

^g^ Households with a high occupancy level are those where the number of people who usually sleep at the dwelling exceeds the number of bedrooms in the dwelling by four.

^h^ A nine-item scale was used to measure the extent to which families have established an environment of cooperation, emotional support and good communication. Ratings from scores provided by carers were summed to produce an overall score that was categorised into quartiles, and labelled ‘poor’, ‘fair’, ‘good’ and ‘very good’.

^i^ Primary carers were asked if any of fourteen major life stress events had occurred in the family in the preceding 12 months. These events included events such as illness, hospitalisation or death of a close family member, family break-up, arrests, job loss and financial difficulties.
